# Metabolomic Profile in Venous Thromboembolism (VTE)

**DOI:** 10.3390/metabo11080495

**Published:** 2021-07-29

**Authors:** Beata Franczyk, Anna Gluba-Brzózka, Janusz Ławiński, Magdalena Rysz-Górzyńska, Jacek Rysz

**Affiliations:** 1 Department of Nephrology, Hypertension and Family Medicine, Medical University of Lodz, 90-549 Lodz, Poland; beata.franczyk-skora@umed.lodz.pl (B.F.); jacek.rysz@umed.lodz.pl (J.R.); 2 Department of Urology, Institute of Medical Sciences, Medical College of Rzeszow University, 35-055 Rzeszow, Poland; janlaw@wp.pl; 3 Department of Ophthalmology and Visual Rehabilitation, Medical University of Lodz, 90-549 Lodz, Poland; mrs-89@o2.pl

**Keywords:** venous thromboembolism, deep vein thrombosis, pulmonary embolism, metabolomics, profiling

## Abstract

Venous thromboembolism (VTE) is a condition comprising deep venous thrombosis (DVT) and pulmonary embolism (PE). The prevalence of this disease is constantly increasing and it is also a chief reason for morbidity. Therefore, the primary prevention of VTE remains a highly important public health issue. At present, its diagnosis generally relies on subjective clinical examination and ultrasound imaging. D-dimer is also used as a biomarker, but it is considered to be poorly specific and only moderately sensitive. There are also no reliable methods that could accurately guide the type of treatment and potentially identify patients who may benefit from more aggressive therapies without the risk of bleeding. The application of metabolomics profiling in the area of vascular diseases may become a turning point in early diagnosis and patient management. Among the most described metabolites possibly related to VTE are carnitine species, glucose, phenylalanine, 3-hydroxybutarate, lactic acid, tryptophan and some monounsaturated and polyunsaturated fatty acids. The cell response to acute PE was suggested to involve the uncoupling between glycolysis and oxidative phosphorylation. Despite technological advancement in the identification of metabolites and their alteration in thrombosis, we still do not understand the mechanisms and pathways responsible for the occurrence of observed alterations.

## 1. Introduction

Venous thromboembolism (VTE) is a condition comprising deep venous thrombosis (DVT) and pulmonary embolism (PE) [1]. In the United States, VTE affects ~300,000–600,000 individuals and it is responsible for approximately 100,000 deaths per year [2]. This disease, whose prevalence is constantly increasing, is also a chief reason for morbidity [2]. Therefore, the primary prevention of VTE remains a highly important public health issue. However, due to the fact that venous thromboembolism is a multifactorial disorder covering complex genetic, metabolic, and environmental interactions, a thorough understanding of disease physiological features is necessary to improve primary prevention. Deep venous thrombosis (DVT) is a frequent cause of hospital-acquired mortality, which can be prevented by providing an early, specific diagnosis [3]. A venous thrombus may develop at any site within the veins, in legs, arms, cerebral or abdominal regions, or others. Many cases of venous thrombosis resolve without complications as a result of spontaneous lysis and recanalization, but about 50% of patients (despite optimal anticoagulant therapy) suffer from post-thrombotic syndrome characterized by pain, swelling, skin changes, and/or venous ulceration. However, in some individuals, the presence of DVT may be associated with severe life-threatening pulmonary embolism when blood clots disrupt and migrate [2,4,5,6]. Apart from the aforementioned, the presence of thrombosis may obstruct blood flow resulting in the occurrence of possibly fatal diseases, such as myocardial infarction, stroke, atherosclerosis and coronary heart disease [7]. The formation of venous thrombus is a complex and dynamic physiological process in which platelets, leukocytes, erythrocytes, and fibrin play a vital role [8]. In the initiation stage of DVT formation, the cross-talk between monocytes, neutrophils, and platelets is necessary. At present, the diagnosis of DVT generally relies on subjective clinical examination and ultrasound imaging [5,9]. A biomarker, D-dimer, is also used, but it is considered to be poorly specific and only moderately sensitive [3,10]. These measurements are unable to estimate the age of the clot, which is important as thrombolysis can be effectively used to lyse the clot only within the first two weeks from the clot development [3]. There are also no reliable methods that could accurately guide the type of treatment and potentially identify patients who may benefit from more aggressive therapies without the risk of bleeding. In turn, the development of pulmonary embolism may lead to pulmonary hypertension and right ventricular failure resulting from pulmonary arterial bed occlusion. In addition, the diagnosis of PE is challenging as a result of non-specific clinical presentation, which leads to relatively high mortality [11].

Many risk factors related to the development of VTE have been established (advanced age, immobility, surgery, and obesity, but also blood vessel injury, inflammation and blood flow), however, most biological mechanisms associated with this disorder remain unsolved [7]. In many patients, genetic or acquired biomarkers or risk factors of venous thromboembolism (VTE) remain unidentified [12]. The susceptibility to VTE has been linked with disturbances within the clotting system, but the results of recent studies utilizing genome-wide association techniques suggested the existence of loci possibly related to VTE prevalence, which are not part of the coagulation cascade [4,13]. Despite the knowledge of risk factors and the implementation of thromboprophylaxis, the prevalence of VTE has remained unchanged over the past decade [14]. The unmet need to find new biomarkers and causal risk factors in these patients has resulted in the use of novel techniques. The application of metabolomics profiling in the area of vascular diseases may become a turning point in early diagnosis and patient management. However, the time after which the metabolites appear in biological samples and they become indicative of diagnosed disease also requires clarification. Therefore, it seems that the application of metabolomic profiles in the diagnosis of disease will require overcoming numerous obstacles before it can be introduced into clinical practice.

Table 1 presence between venous thrombosis and arterial thrombosis.

## 2. Metabolomics and Related Challenges

The term ‘metabolomics’ refers to the comprehensive and systematic profiling of all low molecular weight compounds, which are intermediate or final products of metabolism, generated as a product of biochemical and physiological processes in the body and present in differential abundance in biofluids, cells and tissues [25,26]. Due to the fact that metabolites represent the downstream expression of a genome, transcriptome and proteome, they mirror an individual’s phenotype at the time of sample collection [25,27]. The abundance of these molecules can be affected by the disease, genetics, medications, environmental factors, diet and lifestyle [5]. Metabolites comprise peptides, carbohydrates, lipids, as well as metabolic intermediates, including amino acids, organic acids, and drug metabolites [26]. The identification of metabolic phenotypes can facilitate the understanding of molecular mechanisms underlying the development or progression of some diseases, seen as changes in the metabolome, which reflects the alteration in biochemical signals associated with gene and protein function by the disease [23]. Metabolic characterization of a sample can be performed with the use of two different approaches: targeted or untargeted [5]. The first one involves the analysis of specific metabolites, which are characteristic of the suspected biological pathway in each sample. In turn, during non-targeted analysis, multiple small molecules are screened in order to find alterations in their levels, which enables the identification of novel metabolites and pathways, the clarification of the pathophysiology of the disease, and pinpointing of potential biomarkers, biomarker signatures, and/or therapeutic targets [5]. Metabolomics profiling requires the combined application of high-throughput analytical platforms, such as ultra-performance liquid chromatography-mass spectrometry (UPLC-MS), gas chromatography-mass spectrometry, nuclear magnetic resonance (NMR) spectroscopy and statistical modelling platforms [5,25]. The aforementioned methods enable the collection of complex multidimensional data, which requires subsequent statistical analysis. However, due to the fact that metabolic products display various physicochemical properties and their concentrations in collected samples are different, there is no single analytical technique enabling their complete analysis in a single run [26]. The simplicity of sample collection and the fact that serum and plasma mirror metabolic alterations occurring throughout the body explains why these sources are frequently used for metabolic profiling. However, in some cases, the study of specific tissues may improve the sensitivity and specificity of signature metabolic profiles. The urine may serve as a more appropriate source for the analysis of metabolites that are rapidly cleared from the circulation [26]. 

The study of metabolites can reveal the physiologic state before disease onset as well as the complex interplay between genes, environmental risk factors, and VTE [1]. This technique has been used in the study of cardiovascular disease risk, however, there are just a few publications concerning metabolic profiling in VTE. The differentiation of venous thrombosis may provide an excellent tool enabling the widening of our knowledge on molecular mechanisms of this disease, as well as the identification of new drug target opportunities. 

### Challenges

The field of metabolomics has been recently extensively developed and used for the identification of potential biomarkers for the early diagnosis of diseases. The key to the advancement of medicine is the improvement of our understanding of disease pathophysiology [3]. The use of metabolomics technologies appears to provide us with a tool to approach this goal. However, the application of metabolomics is associated with some challenges that we have to overcome. First of all, due to the fact that this method is tremendously sensitive, even a small change in sample collection or analysis may exert a marked impact on the obtained results [3]. Moreover, it seems that metabolite identification at a single time-point offers only limited data on complex, dynamic disease processes, and therefore serial measurements should be performed to establish the context [28]. Furthermore, a single metabolite can be involved in the development of multiple pathologies, greatly reducing its potential to discriminate a given disease. The profiling of patient samples must be preceded by an in-depth analysis of the patient’s health state. Apart from comorbidities, other confounding factors, such as age, diet, and the individual’s genome and microbiome can also be related to systemic metabolic alterations [29,30]. Moreover, the treatment also alters the metabolic profile. Brunner et al. [31] analysed individual metabolites and groups of metabolites following the administration of unfractionated heparin. They found that heparin administration was associated with measurable changes in peripheral blood metabolite concentrations in samples obtained during cardiac catheterization procedures. This drug induces the release of endothelial and hepatic lipoprotein lipase (LPL), and therefore it could promote the hydrolysis of triglycerides in chylomicrons and very-low-density lipoprotein into two non-esterified fatty acids (NEFAs) and monoacylglycerol [32]. However, the extent to which heparin could affect the metabolite concentrations has not been assessed in patients with DVT or PE. Brunner et al. [31] reported that in patients presenting for cardiac catheterization procedures, only four metabolites (alpha-hydroxybutyrate; ketones, NEFA and triglycerides) differed significantly between the non-heparin and heparin groups. Unfortunately, there are no studies analysing the impact of also other drugs used in DVT and PE patients on metabolomic profile.

The interpretation of obtained data in isolation is difficult, and therefore there is a need for cross-omics studies with validation through different experimental approaches. Information obtained with the use of metabolomics should be analysed in combination with other omics studies in order to allow the understanding of disease-related mechanisms. However, such data is still not available in the current publications. These factors make the establishment of universal reference values, which could be used in diagnostic tests, challenging. The creation of cumulative and reliable databases based on data from large populations studies, such as the human metabolome database, could help to overcome these obstacles. Moreover, performing epidemiological studies could enable the collection of data concerning the distribution of metabolites in different patient populations [3,33]. 

In this review, we would like to summarize the result of metabolic studies performed in animal models of DVT and PE and also sparse data obtained from the analysis of human samples.

## 3. Deep Venous Thrombosis and Pulmonary Embolism

### 3.1. Deep Venous Thrombosis in Animal Studies

#### 3.1.1. Metabolome Profiling of Venous Thrombus and Vein Walls

The results of metabolome profiling in animals demonstrated that levels of several molecules involved in energy metabolism may be used as discriminatory factors between DVT and control animals [5]. Maekawa et al. [34] studied metabolic alterations in venous blood and venous thrombus of rabbits with the use of capillary electrophoresis-time of flight mass spectrometry (CE-TOFMS). Their study demonstrated that lactic acid, glycine, glutamate, cysteine glutathione disulphide, glutamine, and lysine, in descending order were the most abundant metabolites in venous thrombus, and their concentrations were at least 5-fold higher compared to the venous blood. In turn, the concentrations of citric acid, glucose 6-phosphate, nicotinamide adenine dinucleotide phosphate+ (NADP+), tryptophan, and fructose 6-phosphate were considerably decreased in the venous thrombus compared to the venous blood [34]. Fresh venous thrombus of studied rabbits contained different levels of metabolites related to glycolysis, purine and tryptophan metabolism as well as redox reactions compared to venous blood. These differences may be partly associated with various cellular and serum components. The protective role of tryptophan derivative, TD-26 in thrombosis was suggested by Chen et al. [35] who observed that it diminished the death rate from acute PE by 90% and decreased thrombosis weight by 60%. Further experiments revealed that TD-26 inhibited platelet aggregation via blocking the binding of fibrinogen to integrin and lowering protein kinase B phosphorylation in platelet phosphatidylinositol 3-kinase signalling. According to studies, glucose consumption related to erythrocyte metabolism is greater in deoxygenated erythrocytes compared to oxygenated erythrocytes. Moreover, a major transmembrane protein of erythrocytes, band 3, displays a higher affinity for glycolytic enzymes, including glyceraldehyde-3-phosphate dehydrogenase, phosphofructokinase, lactate dehydrogenase and aldolase, resulting in the inhibition of the glycolysis pathway [36]. The competition between deoxyhemoglobin and glycolytic enzymes for the cytoplasmic domain of band 3 results in erythrocyte metabolic modulation under hypoxia [37]. In turn, the rise in the concentration of lactate levels and the reduction of glycolytic metabolite levels were suggested to be associated with boosted glycolysis in intra-thrombus erythrocytes [34]. Maekawa et al. [34] found that platelets were the most abundant cellular component of venous thrombus. It has been suggested that central carbon metabolism in these compounds may contribute to platelet function, which is in agreement with the rise in lactate levels and drop in glycolytic metabolite concentrations in the venous thrombus [38]. In vitro studies have demonstrated that levels of lactic acid, guanine, or AMP can exert an impact on blood coagulation or platelet aggregation. In the study performed by Maekawa et al. [34], lactic acid, which was the most abundant metabolite in the venous blood and thrombus, stimulated (to some extent) whole blood coagulation and hampered platelet aggregation. In turn, Crowell et al. [39] demonstrated that the infusion of lactic acid was associated with significantly decreased whole blood clotting time in vitro and in vivo as the pH became lower. The confirmation that lactic acid, rather than acidaemia, stimulated blood coagulation was derived from a study indicating that the use of hydrochloric acid to adjust the pH level in blood samples from healthy volunteers was associated with impaired whole blood coagulation assessed based on thromboelastography [40]. However, the net effects of lactate on the formation and stabilization of venous thrombosis needs additional studies. Maekawa et al. [34] also indicated higher content of purine metabolites, AMP, hypoxanthine, and guanine in venous thrombus compared with the blood, while adenosine was detectable solely in the venous thrombus. Increased concentrations of adenosine (2.2-fold rise) was also reported in the vein wall in other mouse models of deep vein thrombosis, while its levels in serum were found to be considerably decreased (9.5-fold) in comparison to control vein wall and serum [5]. The amount of adenosine, produced as a result of intracellular or extracellular metabolism of adenine nucleotides, can be affected by numerous factors, including hypoxia, stress, cell damage [41,42]. The involvement of adenosine in thrombosis has been described in numerous publications [5]. It was found to prevent platelet activation and aggregation, and inhibit tissue factor in endothelial cells [43,44,45]. According to Sung et al. [5], decreased serum adenosine levels in thrombosis could be associated with enhanced uptake by endothelial cells accompanied by its diminished intracellular catabolism. However, it is difficult to clarify why the levels of adenosine in the vein wall are increased as opposed to serum. Authors suggested that this effect might be related to hypoxia-induced by IVC ligation [5]. The observation of altered purine metabolism in DVT (Maekawa et al. [34]) is in agreement with the results of other studies. Both the uptake and release of purines by erythrocytes are influenced by changes in pH, inorganic phosphate, and oxygen concentration. The authors suggested that the accumulation of purine metabolites in the venous thrombus may be associated with purine nucleotides catabolism, elevated lactate level and diminished oxygen supply in the intra-thrombus environment [34]. Maekawa et al. [34] found AMP accumulation in the venous thrombus. Other studies indicated that AMP inhibited platelet adhesion and aggregation via the adenosine A2 receptor and the increase in cyclic AMP level and destabilized aggregated platelets following the formation of venous thrombus [46]. Moreover, AMP repressed P-selectin expression and GPIIb/IIIa activation in a concentration-dependent manner. In the study based on the rabbit model, the concentrations of tryptophan metabolites, including serotonin and kynurenine (Kyn) pathway metabolites, were also found to be higher, while the level of tryptophan was reduced in the venous thrombus compared with the blood [34]. According to Wang et al. [47], the kynurenine pathway is the chief pathway for tryptophan metabolism, which contributes to several essential biological processes. They suggested that Kyn might be used as a novel marker of immune activation in early atherosclerosis. Moreover, 3-hydroxykynurenine and quinolinic acid were found to positively correlated with inflammation, oxidative stress, endothelial dysfunction, and carotid artery intima-media thickness values. In turn, the enzyme involved in the catabolism of tryptophan to kynurenine-indoleamine 2, 3-dioxygenase 1 was found to stimulate thrombus formation through the upregulation of tissue factor in activated macrophages [48]. The exact role of kynurenine metabolites in the pathophysiology of DVT remains unravelled; it appears that they may exert an impact on thrombus formation via the modulation of the thrombotic or reparative function of monocytes and macrophages [34]. In another study, metabolome analysis focused on vein walls and blood samples of a murine inferior vena cava ligation DVT model compared to sham surgery controls [5]. In this analysis, alterations in energy, lipids and purine metabolism, including higher levels of acetyl carnitine, adenosine and ceramide in the vein wall of DVT animals, were observed. In turn, Sung et al. [5] found significantly higher levels of ceramide and SMs within the vein wall of DVT mice [5].

#### 3.1.2. Metabolome Profiling of Whole Blood, Serum

Maekawa et al. [34] observed serum metabolite alterations including changes in purine metabolism which, in the opinion of the author, were indicative of metabolic shifts in intra-thrombus cells of patients with VTE. The alteration in the level of three different metabolic products was observed by Obi et al. [23]. In their study, blood analysis with NMR metabolomics in experimental mice with thrombus induced using the electrolytic inferior vena cava revealed that the levels of three metabolically related compounds: glutamine, phenylalanine, and proline were higher in the blood of old animals compared with young ones. Concentrations of these three metabolites correlated with vein wall weight and vein wall P-selectin levels. This study indicates that age may affect the physiology and the course of venous thrombosis. The age-related rise in glutamine, phenylalanine, and proline was suggested to be the consequence of the decreased activity of enzymes catalysing their conversion and this reduction appears to be linked with higher oxidative stress observed in ageing individuals [49,50,51,52]. In general, Obi et al. [23] suggested that physiological and metabolic responses contributing to the aetiology of venous thrombi may be different in young and old animals. Ageing-related enhanced oxidative stress can result in the inhibition of antioxidant defence mechanisms as well as the inactivation of enzymes and proteins vital for normal vascular function, which translates into deleterious effects on the vascular system [23,53]. Therefore, it appears that oxidative stress related to ageing may also contribute to the metabolic mechanism of venous thrombosis [23]. Animal studies revealed that the accumulation of TMAO in the serum of mice colonized with TMA-producing species aggravated the metabolic disease, induced epigenetic changes and boosted platelet reactivity and thrombosis formation [54,55,56]. TMAO-related enhanced risk of thrombosis was suggested to be associated with the direct stimulation of platelet responsiveness to multiple agonists, the modulation of platelet function and increased Ca^2+^ release from intracellular stores [57]. Numerous studies have indicated that the formation of platelet thrombi is fundamental for the development of most vascular ischemic events and that platelet hyperreactivity precedes the occurrence of vascular thrombotic diseases [58]. TMAO has also been demonstrated to stimulate the progression of atherosclerosis via the inhibition of reverse cholesterol transport, which results in the accumulation of lipids in vessel wall macrophages [58,59]. In another study, metabolome analysis focused on vein walls and blood samples of a murine inferior vena cava ligation DVT model compared to sham surgery controls [5]. In this analysis, blood concentrations of adenosine, adenine, and intermediate molecules of tricarboxylic acid (citrate, succinate and fumarate) were reduced, while L-carnitine, sphingomyelins, phosphatidylcholines and triglycerides were elevated. These findings indicate compromised tricarboxylic acid (TCA) turnover probably resulting from diminished availability of acetyl-CoA. Due to the fact that no marked changes in the rate of glycolysis were found, the authors suggested that decreased availability of acetyl-CoA could be related to disturbed fatty acid metabolism involving carnitine species [5]. Carnitines are involved in the conversion of activated FFAs to acylcarnitine and the transport of acylcarnitines into mitochondria [5]. In the subsequent step, acylcarnitines are transformed into acyl CoA, which is then subject to β-oxidation to form the acetyl-CoA that is involved in the TCA pathway [60]. The impact of disturbed carnitine metabolic pathway remains not fully elucidated, however, some studies indicated its relationship with various disease states [61,62,63]. The finding that carnitine levels were elevated in serum from DVT mice may suggest a disruption in the pathway converting carnitines to acylcarnitines, leading to the accumulation of carnitines and decreased formation of acylcarnitines in these animals [5]. Correlation analysis performed by Sung et al. [5] demonstrated a strong association between triglycerides (TG) and acetyl carnitine, but a weak association with carnitine, therefore it cannot be confirmed whether or not disturbances within the pathway converting carnitines to acylcarnitines are related to the reduced level of TGs observed in their study. Levels of sphingomyelins (SM), belonging to the sphingolipid family, were also found to be affected by the presence of DVT in mice [5]. Sphingolipids, including ceramide and sphingosine-1-phosphate (S1P), are bioactive lipids with diverse functional and structural roles ranging from apoptosis, cell senescence, cell migration, and cell survival [64]. Some studies indicated that compromised sphingolipid metabolism may be associated with the presence of cardiovascular disease and inflammatory processes [65,66]. Moreover, it was found that oxidized LDL, growth factors or cytokines present in the atherogenic lesion can promote sphingomyelin hydrolysis and generation of ceramide and other metabolites, such as sphingosine-1-phosphate [65]. According to studies, ceramides stimulate both the release of Weibel-Palade bodies from endothelial cells and E-selectin dependent leucocyte adhesion onto endothelial cells [67,68]. Selectins were demonstrated to stimulate inflammation and venous thrombosis [69,70]. The results of other studies indicate the involvement of ceramides and their products in coagulation. Based on their finding, Sung et al. [5] suggested that increased levels of ceramide in the vein wall of DVT mice could trigger thrombogenesis via the stimulation of endothelial cell activation and thrombus generation. However, subsequent studies are required to provide a detailed explanation of this mechanism.

#### 3.1.3. Metabolome Profiling of Urine

In turn, Cao et al. [71] analysed the urinary metabolic profile in a rat model of deep venous thrombosis obtained by ligation of the inferior vena cava. They observed that such profiles considerably differed between DVT, sham and control rats. The urine of DVT rats contained elevated levels of leucine, glutamine, creatine, creatinine and sucrose compared to the control group. Moreover, the levels of 3-hydroxybutyrate, lactate, acetone, α-oxoglutarate, citrate and hippurate were found to be decreased in the urine of DVT animals [71]. The differences in metabolite profile may serve as a source of potential biomarkers enabling early detection of DVT and the monitoring of disease progression as well as treatment outcomes. 

### 3.2. Pulmonary Embolism in Animal Studies

**Figure 1:**
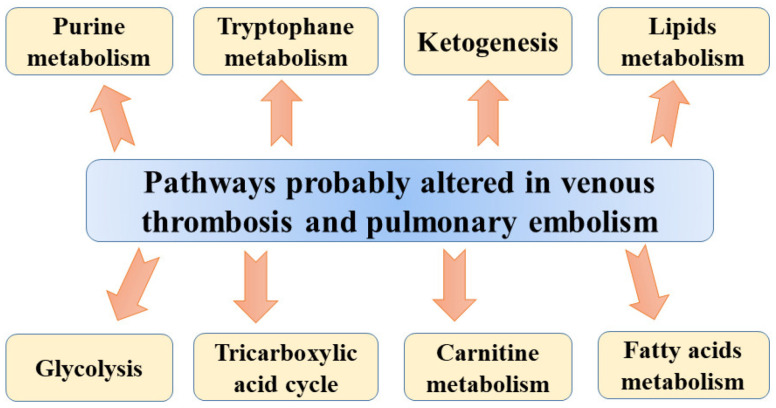
Suggested pathways altered in venous thrombosis and pulmonary embolism.

Metabolomic studies were also used in pulmonary embolism models. Bujak et al. [11], analysing metabolic plasma profiles obtained from a pig model of pulmonary embolism, found altered levels of many metabolites involved in glycolysis, intermediates of tricarboxylic acid (TCA) cycle, lipid metabolism, and ketone bodies. The differences in concentrations of intermediates of the TCA cycle, carnitine species, and triglycerides (TGs) may suggest a global change in energy metabolism in DVT. The TCA cycle is necessary for converting acetyl-CoA into few intermediates, producing at the same time CO_2_ and reduced forms of nicotinamide adenine dinucleotide (NADH) or flavin adenine dinucleotide (FADH_2_). In subsequent steps, reduced coenzyme groups are used by mitochondria to synthesize ATP via the oxidative phosphorylation pathway [11]. The accumulation of TCA cycle intermediates was reported in a hypoxic state when the oxidative phosphorylation rate is decreased as a result of oxygen shortages. Higher concentrations of citrate, malate, fumarate, and α-ketoglutarate observed in pigs’ plasma could be associated with diminished TCA cycle turnover. The lower rate of the TCA cycle may also result in the accumulation of acetyl-CoA, which is used for ketogenesis giving rise to the elevated concentrations of β-hydroxybutyrate (β-HB) and acetoacetate (ketone bodies) following acute PE. According to Bujak et al. [11], altered levels of ketone bodies could be associated with hypoxia-mediated alterations resulting from acute PE. They also found marked differences in levels of pyruvate and lactate between plasma obtained from animals before injection of microspheres and after 1 h of PE. These alterations can be associated with the aforementioned protective mechanism of shifting glucose metabolism towards the generation of ATP and prevention of mitochondrial ROS production. The results of other studies confirmed that the presence of hypoxia can shift ATP generation toward glycolysis (Warburg effect) resulting in the cytosolic accumulation of pyruvate, which could be subsequently converted to lactate by lactate dehydrogenase [72,73]. Moreover, enhanced consumption of glutathione during hypoxia can be associated with higher levels of α-hydroxybutyrate (α-HB) and pyroglutamate in PE animals. The glycolytic shift observed by Bujak et al. [11] results from the stabilization of the transcriptional regulator of the hypoxic response, hypoxia-inducible factor (HIF), which regulates many enzymes involved in glycolysis, leading to the stimulation of pyruvate dehydrogenase kinase and lactate dehydrogenase but also the inhibition of pyruvate dehydrogenase [74,75]. Apart from vasoconstriction, increased vascular cell proliferation, and resistance to apoptosis, TCA is also involved in the stabilization of HIF [11]. Finally, the energy imbalance observed by Bujak et al. [11] results in abnormalities in lipid metabolism, including the rise in glycerol and the free fatty acids (FFA) docosatetraenoic and docosapentaenoic acids and palmitic and oleic acids. These changes may stem from enhanced lipolysis as other studies also reported the induction of lipolysis in hypoxia [76]. Another explanation of altered free fatty acids level following PE may be related to increased activity of hypoxia-modulated phospholipase A2 (PLA2), which converts phospholipids into FFA, eicosanoids (e.g., oxylipins), and lysophospholipids [77]. The level of oxylipins was found to be significantly altered in the pig model of PE [11]. Apart from these molecules, lower sphingomyelin and ceramide-1-phosphate (Cer-1-P) and higher sphingosine were observed after acute PE in pigs. Sphingolipids, which are essential lipid constituents of membranes in eukaryotes were also found to be engaged in cell growth, apoptosis, signal transduction, and recognition [78]. In addition, Cer-1-P (formed via direct phosphorylation of ceramide by ceramide kinase (CERK)) plays an important role in cell growth and survival, but it is also a vital mediator of the inflammatory response via the stimulation of cytosolic phospholipase A2 (cPLA2) [79]. On the basis of obtained results, Bujak et al. [11] suggested that the cell response to acute PE involves the uncoupling between glycolysis and oxidative phosphorylation. However, the finding needs confirmation in human studies due to the fact in that study, experimental PE was obtained using injected polydextrane microspheres, not thrombi. Suggested pathways altered in venous thrombosis and pulmonary embolism are presented in Figure 1.

### 3.3. Effects of Anti-Thrombotic Drug Compounds on the Metabolome Profile

In turn, Ma et al. [7] analysed the effect of aspirin eugenol ester (AEE) on metabolite abundances in carrageenan-induced thrombosis in rats. This study demonstrated that carrageenan-induced thrombosis decreased tryptophan and methionine levels, which may suggest that it had an impact on the metabolism of these amino acids as well as a reduced concentration of linoleic acid. This finding is in agreement with the results of other studies showing the involvement of methionine in the pathogenesis of venous thrombosis since it exerts an impact on redox reactions regulating physiologic responses related to thrombosis, including platelet aggregation, cell adhesion, inflammation and vasoconstriction [80,81]. Low plasma concentrations of methionine are considered a thrombosis risk factor [82]. Following the administration of AEE, fifteen plasma metabolites related to thrombosis were found to be altered. They were mainly related to fatty acid metabolism, energy metabolism and amino acid metabolism. AEE therapy markedly increased the levels of linoleic acid and arachidonic acid, which may suggest that alteration of fatty acid metabolism could exert a cardioprotective effect [7]. Moreover, AEE decreased allantoin levels. According to studies, allantoin is a sensitive marker of oxidative stress and therefore its higher level in rats with thrombosis may be indicative of oxidative impairment [83]. AEE-related reduction in allantoin implies treatment-related improvement of oxidative damage. Ma et al. [7] also found that AEE diminished the level of carnitine, which could ameliorate energy impairment. Interestingly, they observed that acetylsalicylic acid (ASA), eugenol and aspirin eugenol ester exerted various effects on some metabolites (including allantoin, carnitine and citric acid), which shows their regulatory effects on thrombosis. 

### 3.4. Deep Venous Thrombosis in Human Studies

There are just a few human studies of the metabolite profile of thrombosis. The application of untargeted metabolomics in the search for biomarkers of VTE in 40 male idiopathic adult VTE cases and 40 age-matched male controls resulted in the identification of 9400 metabolic features, 257 of which markedly differed between controls and patients with VTE in terms of serum concentrations [12]. Furthermore, it was found that nearly 90% of these 257 traits were metabolites related to warfarin. Following the elimination of warfarin-associated metabolites, the authors observed that the level of 6 metabolites was markedly higher, while 22 were significantly lower in patients with VTE compared with controls [12]. They reported decreased plasma levels of acylcarnitines in patients with venous thromboembolism (VTE) compared to healthy individuals [12]. Long-chain acylcarnitines (AC), palmitoleoyl carnitine, and decenoyl carnitine, were identified in the performed untargeted metabolomics as VTE-associated metabolites. Patients with low levels of these two AC (<10th percentile of control) had increased VTE risk. Detailed analysis revealed considerably decreased plasma levels of 10:1-AC, 12:0-AC, 12:2-AC, 18:1-AC, 18:2-AC, and total long-chain ACs (n ≥ 10) compared to matched controls without VTE (*p* = 0.01, 0.004, 0.001, 0.04, 0.02, and 0.03, respectively). AC circulating in plasma plays a vital role in mitochondrial energy metabolism [12,84]. The study of the mechanism underlying anticoagulant activity of long-chain-ACs demonstrated that ACs were able to inhibit factor Xa-initiated clotting processes, and this effect was found to be concentration-dependent and acyl chain length-dependent (ACs with longer acyl chains were more powerful anticoagulants compared to ACs with shorter acyl chains). To display anticoagulant properties, 16:0-AC did not require factor Va nor phospholipid. However, it was observed that ACs did not interact with active sites of factor Xa or thrombin and thus they did not inhibit the thrombin-induced clotting process; therefore, the authors implied that ACs might act solely on prothrombinase complex disrupting interactions between factor Xa and prothrombin [12]. Moreover, they observed that 16:0-AC bound to factor Xa and DG-factor Xa with similar affinities repressing both Gla-DG-prothrombin activation by factor Xa and prothrombin activation by DG-factor Xa [12]. Some studies suggested that the concentrations of ACs might be increased in certain metabolic conditions [85,86]. Deguchi et al. [12] suggested that the anticoagulant effect of long-chain acylcarnitines could be physiologically relevant. Significantly diminished levels of many long-chain ACs in VTE patients (values below the 20th percentile of controls) could suggest that some patients had the deficiency of plasma anticoagulant lipids resulting from a shortage of multiple long-chain ACs. Similarly, Jiang et al. [1] who studied 240 incident VTE cases (including 125 incident PE cases) and 6963 controls revealed that C5 carnitine was significantly associated with incident VTE. They demonstrated that diacylglycerols were increased in both VTE and PE patients, but their exact role in the pathomechanism of VTE is unknown. The enrichment of diacylglycerols in VTE and PE may mirror the intermediate effect of triacylglycerols. Despite the fact that no sound epidemiological evidence confirms the relationship between diacylglycerols and risk of incident VTE, the results of animal studies revealed that diacylglycerol-enriched diet repressed arterial thrombus formation in ApoE and low-density lipoprotein receptor double-negative male mice [87]. This effect could be driven by the protection of vascular endothelium from injury and diminished serum levels of low-density lipoprotein cholesterol [88]. Jiang et al. [1] hypothesized that both long- and short-chain carnitines may cooperate as a part of the haemostatic system and the pathophysiological characteristics of VTE. Apart from carnitines, they also observed a nominal association between triacylglycerols, phosphatidylethanolamines, and amino acids (tryptophan) and the risk of VTE and PE. However, after the adjustment for BMI, metabolites no longer remained strongly associated with this disease. The involvement of elevated circulating triacylglycerols (>1.05 mmol/L) in the enhanced risk of VTE was also demonstrated in a study of 477 postmenopausal female VTE cases and 1986 sex, age-matched controls, as well as in a small case-control study [89,90]. It was suggested that this elevated risk could be related to a reduced ratio of activated protein C43 and elevated concentrations of coagulant factors [91]. Other studies indicated the presence of antibodies against phosphatidylethanolamines in patients with thromboembolic events, including PE, and stroke [92]. Sanmarco et al. [93] confirmed in a population-based retrospective case-control study of patients with VTE that anti-phosphatidylethanolamine antibodies enhanced the risk of thrombosis four-fold. The aforementioned findings imply that circulating metabolites, such as short-chain carnitine and possibly diacylglycerols, seem to be related to the risk of incident VTE. In addition, Fraser et al. [25] used the metabolomics approach (three untargeted liquid chromatography-mass spectrometry analyses in positive and negative ionization modes) to analyse polar and semi-polar metabolites, as well as lipid profiles in plasma collected from patients with post-VTE compared to that of VTE free patients. Their study demonstrated differential expression of twenty-five metabolic and lipid blocks between these two groups. Sixteen out of 21 metabolites selected to predict the thrombosis could be assigned into 14 biological functions, including oxidative stress/inflammation, cell defence system, cell signalling, primary metabolism, metabolic control and dysregulation, gut microbiota metabolism of tryptophan and vascular function [25]. Lipids cluster 1 (rich in triglycerides and phosphatidylcholines, with lipid species primarily containing esterified long-chain polyunsaturated fatty acids of n-6 and n-3 series) seemed to be fundamental in coordinating the specific VTE metabolic response. Twelve lipid species were identified as biomarkers of past VTE. Numerous studies demonstrated that high consumption of 2 n-3 long-chain polyunsaturated fatty acids was associated with antithrombotic effects and decreased VTE risk [94,95,96]. However, the results of Fraser et al. [25] showed the relationship between n-3 long-chain fatty acid lipids levels and VTE in the studied group. The role of these lipids in the development of VTE remains unknown; however, the authors suggested that the aforementioned lipids may interact or control various functions in patients with a previous VTE. Similarly to the results of animal studies, Fraser et al. [25] also reported a relationship between tryptophan metabolism and gut microbiota metabolism. Other studies also found that the modulation of tryptophan metabolism affects VTE via the activation of the aryl hydrocarbon receptor pathway but also via the generation of prothrombotic TMAO [97,98,99,100]. Indeed, in the described study, TMAO concentrations were two times higher in the plasma of incident (and recurrent) VTE patients compared with healthy controls; however, due to its variability, it cannot be used as a biomarker [25]. Moreover, Fraser et al. [25] observed that 3 months after the occurrence of VTE, more than 70% of the VTE populations remained different from the healthy controls. However, the authors stated that none of the reported biomarkers could robustly discriminate the cases from the healthy control individuals; therefore, they suggested the use of multiple biomarkers [25]. Such a combination of biomarkers proved useful in the prediction of incident VTE in the majority of the relapsed patients. The authors suggested that the VTE permanent metabolic phenotype was independent of the number of events [25].

### 3.5. Pulmonary Embolism in Human Studies

The study of patients with different severities of pulmonary embolism (PE) has revealed the presence of considerable differences in the tricarboxylic acid cycle, fatty acid, and purine metabolite pathway between low- and intermediate/high-risk PE patients [101]. Apart from being a marker of venous thrombosis, lactate was suggested to have prognostic potential in patients with pulmonary embolism. In addition, the results of Thrombo-Embolism Lactate Outcome Study Patients demonstrated that the level of lactate exceeding or equal to 2 mmol/L was associated with greater mortality (17.3%; 95% Cl 11.9% to 20%) compared to patients with lower levels (1.6%; 95% CI 0.8% to 1.9%) and all-cause death (hazard ratio 11.67; 95% CI 3.32 to 41.03) [102]. Zeleznik et al. [101] reported significantly different levels of acylcarnitines between low-risk and intermediate/high-risk PE patients, as well as acylcholines between intermediate and high-risk PE patients. These observations are in agreement with the results of other studies (mentioned above), which indicated diminished levels of acylcarnitines in VTE cases compared to controls. Therefore, it appears that acylcarnitines may be related to both risk of incident VTE and PE severity, however, the exact role of these metabolites in VTE pathophysiology requires clarification. Moreover, Zeleznik et al. [101] based on the finding that short and medium-chain acylcarnitines (which differed between low-risk PE and intermediate/high-risk PE) strongly correlated with each other, suggested that they may play role in PE severity. They also demonstrated increased levels of hypoxanthine and xanthosine in patients with intermediate/high risk of pulmonary embolism compared to the low-risk PE group. Additionally, they found lower levels of haptoglobin in high-risk PE patients compared to less severe PE. The down-regulation of haemoglobin metabolism and haptoglobin may play an essential role in the physiology of severe PE and it may enable the identification of patients at risk for adverse outcomes [101]. Table 2 summarizes the results of the above studies concerning alterations in metabolite levels in venous thrombosis and pulmonary embolism.

## 4. Conclusions

The complexity of the thrombosis phenotype could be associated with complex interactions of genetic, environmental and acquired factors, which contribute to disease susceptibility. There is still a long road ahead before we will be able to understand the complicated pathomechanisms of diseases. The utility of high-throughput “omics” in biological systems is great; however, there are still many obstacles to overcome. If we do this, such tools will help to decipher the complexity of human pathology, which in consequence will facilitate the diagnosis and treatment of patients. Among the most described metabolites possibly related to VTE, there are carnitine species, glucose, phenylalanine, 3-hydroxybutarate, lactic acid, tryptophan and some monounsaturated and polyunsaturated fatty acids, which accounts for energetic metabolism. In addition, some specific metabolic alterations have been reported, including TMAO for arterial thrombosis and AC for venous thrombosis. In turn, cell response to acute PE was suggested to involve the uncoupling between glycolysis and oxidative phosphorylation. Despite technological advancement in the identification of metabolites and their alteration in thrombosis, we still do not understand the mechanisms and pathways responsible for the occurrence of observed alterations. Therefore, drawing conclusions concerning various metabolites involved in the development of thrombosis must be preceded by large studies performed with the use of high-throughput analytical techniques and the analysis of their possible role in this disease process.

## Figures and Tables

**Table 1:** Differences between venous thrombosis and arterial thrombosis.

Differences	Venous Thrombosis	Ref	Arterial Thrombosis	Ref
Causes	The occlusion of blood flow of the lower limbs	[15]	Arterial occlusion, typically triggered by erosion/rupture of an atherosclerotic plaque, or as a result of the embolization of a thrombus in the heart or other arteries.	[16,17,18]
Most common consequences	Acute symptoms: swelling and pain. The blood clot can disrupt and migrate causing PE.	[15]	Local tissue ischemia, and ischemic heart and ischemic stroke.	[18,19]
Examples of alterations in metabolomic serum profile	Altered levels of triacylglycerols, phosphatidylethanolamines, and amino acids (tryptophan)	[1]	Altered levels of xanthine and ascorbate (possible markers of atherosclerotic plaque formation). Altered levels of HDL, choline, taurine, glycine and glucose (specific biomarkers of the initial state of the disease).	[20]
	Reduced levels of adenosine, adenine, and intermediate molecules of the tricarboxylic acid (citrate, succinate and fumarate). In creased levels of L-carnitine, sphingomyelins, phosphatidylcholines and triglycerides.	[5]	Methionine, alanine and valine (disease progression).	[21]
	Altered levels of acylcarnitines (palmitoleoyl carnitine, and decanoyl carnitine)	[15]	Altered levels of citric acid, 4-hydroxyproline, aspartic acid, and fructose, L-alanine, L-arabitol, scyllo-inositol, 2-hydroxyphenilacetic acid, 3-hydroxybutiryc acid and N-acetylneuraminic acid, short-chain dicarboxylacylcarnitine and TMAO.	[22]
	Increased levels of glutamine, phenylalanine, and proline	[23]	Altered levels of phenylalanine and monounsaturated fatty acids.	[24]
	Altered levels of betaine and/or trimethylamine N-oxide (TMAO). Altered levels of n-3 long-chain fatty acid lipids.	[25]		

**Table 2:** Alterations in metabolites levels in venous thrombosis and pulmonary embolism.

Venous Thrombosis				
Metabolite Type	Human Studies	Ref	Animal Studies	Ref
Central carbon metabolites (lactic acid, citric acid, glucose 6-phosphate)	No data found	-	Sample: Rabbit venous blood and jugular venous thrombus Results: ↑ lactic acid and ↓ glycolytic metabolite levels (citrate acid, glucose 6-phosphate) in VT vs. VB (*p* < 0.05)– possibly related to enhanced glycolysis in intrathrombus erythrocytes	[34]
			Sample: Serum from DVT mice Results: ↓ tricyclic acid cycle (TCA) intermediates: citrate (1.5 times), succinate (2.3 times), and fumarate (2.8 times)	[5]
Purine nucleotides and their metabolites (hypoxanthine, guanine, AMP, guanine monophosphate (GMP)	No data found	-	Sample: Rabbit venous blood and jugular venous thrombus Results: ↑ levels of AMP, GMP, hypoxanthine, and guanine levels in VT vs. VB (*p* < 0.05).	[34]
		-	Sample: Serum from DVT mice Results: ↓ adenosine (9.6 fold in DVT), adenine (10.6 fold) Sample: Vein wall from DVT mice Results: ↑ adenosine in vein wall (2.2 fold)	[5]
Tryptophan and its metabolites (serotonin, 3-hydroxykynurenine, tryptophan)	No data found	-	Sample: Rabbit venous blood and jugular venous thrombus Results: ↑ levels of tryptophan metabolites (serotonin and kynurenine pathway metabolites) in VT vs. VB (*p* < 0.05). ↓ tryptophan level in VT (*p* < 0.05).	[34]
Other amino acids	No data found	-	Sample: Mice model of VT-whole blood Results: ↑ glutamine, phenylalanine, and proline in old mice vs. young mice	[23]
Lipid metabolism, lipids, fatty acids (choline, ethanolamine phosphate, L-carnitine, sphingomyelins)	Sample: Idiopathic adult VTE cases (plasma) Results: ↓ long-chain ACs (10:1, 12:0, 12:2, 18:1, and 18:2) (*p* = 0.01, 0.004, 0.001, 0.04, and 0.02, respectively)	[12]	Sample: Rabbit venous blood and jugular venous thrombus Results: ↑ choline levels in VT vs. VB (*p* < 0.05).	[34]
	Sample: Adults with incident VTE cases (blood) Results: altered levels of 12 diacylglycerols, 42 triacylglycerols, 23 phosphatidylethanolamines, 36 phosphatidylcholines, 12 cholesterol esters, 7 sphingomyelins, 9 lysophosphatidylcholines, 6 lysophosphatidylethanolamines, and 22 carnitines	[1]	Sample: Serum from DVT mice Results: ↑ levels of L-carnitine (67.0 fold change), sphingomyelins (1.5 fold), phosphatidylcholines, and triglycerides (1.8 fold) Sample: vein wall of DVT mice Results: ↑ levels of ceramide and sphingomyelins	[5]
	Sample: Fasting plasma samples from patients with VTE Results: ↑ levels of phosphatidylcholines and triglycerides containing fatty acyl moieties composed of long-chain polyunsaturated fatty acids of both the n-6 and n-3 series	[25]		
Other	Sample: Fasting plasma samples from patients with VTE Results: ↑ TMAO levels (2 fold)	[25]	Sample: Rabbit venous blood and jugular venous thrombus Results: ↑ choline levels in VT vs. VB (*p* < 0.05).	[34]
**Pulmonary Embolism**				
Central carbon metabolites (lactic acid, citric acid, glucose 6-phosphate)	Sample: Patients with low-risk PE vs. intermediate/high-risk PE Results: ↓alpha-ketoglutarate, malate, isocitrate, fumarate and cis-aconitate in high-risk patients	[101]	Sample: Pig model of pulmonary embolism (serum) Results: ↑ citrate, malate, fumarate, and α-ketoglutarate-associated with a reduced TCA cycle turnover ↑ pyruvate and lactate-shift in glucose metabolism, beneficial for the generation of ATP and the prevention of mitochondrial ROS production	[11]
Purine nucleotides and their metabolites (hypoxanthine, guanine, AMP, guanine monophosphate (GMP)	Sample: Patients with low-risk PE vs. intermediate/high-risk PE Results: ↑ xanthosine and hypoxanthine in intermediate/high-risk PE	[101]	No data found	
Lipid metabolism, lipids, fatty acids (choline, ethanolamine phosphate, L-carnitine, sphingomyelins)	Sample: Adults with incident VTE cases (blood) Results: 12 diacylglycerols, 42 triacylglycerols, 23 phosphatidylethanolamines, 36 phosphatidylcholines, 12 cholesterol esters, 7 sphingomyelins, 9 lysophosphatidylcholines, 6 lysophosphatidylethanolamines, and 22 carnitines	[1]	Sample: Pig model of pulmonary embolism (serum) Results: ↑ free fatty acids (FFA), such as docosatetraenoic and docosapentaenoic acids, as well as palmitic and oleic acids-increased lipolysis ↑ leukotriene C4 and ↓ in dihydroxyoctadecadienoic acid, hydroxyoctadecenoic acid, and oxoheptadecatrienoic acid ↓ sphingomyelin and ceramide-1-phosphate and ↑ sphingosine	[11]
	Sample: Patients with low-risk PE vs. intermediate/high-risk PE Results: Enriched fatty acid metabolism (Acyl Carnitine) pathway ↓ arachidonoylcholine, oleoylcholine, palmitoylcholine, docosahexaenoylcholine and dihomo-linolenoyl-choline in the intermediate/high-risk group	[101]	No data found	
Other	No data found		Sample: Pig model of pulmonary embolism (serum) Results: ↑ β-hydroxybutyrate (β-HB) and acetoacetate (ketone bodies) -probably due to hypoxia-mediated alterations	[11]
